# “The State of the Union”: Current and Future Perspectives on Patient-Centric Care for Primary Immunodeficiencies and Immune Dysregulatory Diseases

**DOI:** 10.3389/fimmu.2019.01783

**Published:** 2019-07-26

**Authors:** Nicholas L. Hartog, Kelli W. Williams, Roshini S. Abraham

**Affiliations:** ^1^Division of Allergy and Immunology, Michigan State College of Human Medicine, Grand Rapids, MI, United States; ^2^Spectrum Health Helen DeVos Children's Hospital, Grand Rapids, MI, United States; ^3^Division of Pediatric Pulmonology, Sleep Medicine, Allergy and Immunology, Department of Pediatrics, Medical University of South Carolina, Charleston, SC, United States; ^4^Department of Pathology and Laboratory Medicine, Nationwide Children's Hospital, Columbus, OH, United States

**Keywords:** primary immunodeficiencies (PI), patient—doctor relationship, specialty, alliance, global healthcare, immunoglobulin replacement therapy (IGRT), newborn screen (NBS)

The number of molecular defects associated with inborn errors of immunity—or primary immunodeficiency (PI, this includes both genetic immunodeficiencies and immune dysregulatory disorders)—is rapidly expanding, and as of February 2017, there were 354 specific gene defects identified and classified in the International Union of Immunological Societies (IUIS) most recent classification (1). In September 2016, the continental societies for primary immunodeficiencies and immune dysregulatory diseases (PIDDs) came together to form a new alliance, which was christened the International Alliance of Primary Immunodeficiencies Societies (IAPIDS) (2). This alliance has brought the key continental societies into closer union in developing joint educational programs, and facilitating a global approach to the diagnosis, management, and treatment of these rare disorders. While these diseases are still considered rare or orphan diseases individually; collectively, they account for a frequency of ~1:1,200, based on a population prevalence study from over a decade ago (3). In recognition of World PI Week 2019 dedicated to raising worldwide awareness of primary immunodeficiencies and immune dysregulatory disorders, it is useful to take stock of where we are now, globally, in terms of disseminating knowledge, and improving accessibility to diagnosis and appropriate treatment. Since 2010, the World PI Week has brought together medical specialists and healthcare professional organizations (e.g., CIS, ESID, LASID, ASID, APSID, and others), patient organizations (e.g., Jeffrey Modell Foundation, the Immune Deficiency Foundation, IPOPI, and others), as well as industry stakeholders from across the continents to stimulate efforts to improve the recognition, diagnosis, treatment, and quality of life of people with primary immunodeficiency worldwide.

## Patient-Centricity in Primary Immunodeficiency Care

As a United Nations Sustainable Development Goal under “Ensure healthy lives and promote well-being for all at all ages” and a Healthy People 2020 goal, improving access to comprehensive, quality health care services for all patients has become a focal point of discussion and action for many global health care systems. As a result, patient-centered care models have grown in popularity. Patient-centered care promotes collaboration between patients, families, and multi-specialty physician and allied health providers to work together to make an individualized and comprehensive health care plan. In many US hospitals and medical centers, patients now have open access to their personal health information and can easily communicate with providers. Benefits of patient-centered care include shared decision-making, improved health outcomes, and improved patient satisfaction (4, 5). Patients with PI deserve the same access and benefits. Therefore, there needs to be a change in the paradigm of healthcare delivery toward patient-centric health care systems for people with PI across the globe, who face lifelong treatment for these complex diseases.

The primary challenge for most patients with PI is that there is often a prolonged diagnostic odyssey before an accurate diagnosis is achieved, and a rational and personalized treatment plan is developed. As a result, they often visit multiple physicians and health care systems, both for outpatient and inpatient care. During this journey, both patients and caregivers become very familiar with the complexities of their disease, and the limitations and benefits of the healthcare system they encounter, which encompasses not only the medical aspects, but also insurance and social support. Out of necessity, patients and family members have learned to be their own advocates, which is essential to successful medical management in this era of fast-paced medicine. The nature of PI is such that it can sometimes result in discontinuous care, especially during the early stages where there may not be a clear diagnosis or management plan established, or if a patient and/or family is not compliant in their response to medical advice. It is the task of the physician provider and support team in the healthcare institution to ensure that all patients, wherever they may be on the spectrum of their involvement in their own medical care, are adequately educated and included in medical decision-making. In the internet era, where information and mis-information abound, the task of patient education takes on a new dimension. Some patients may be articulate and well-prepared for their physician/medical encounter with lists of questions, potential diagnoses and requests for laboratory testing (including genetic testing). Other patients may be resigned or overwhelmed at navigating a bureaucratic and layered medical system. As medical providers, whatever the role might be, we have to accept these challenges as opportunities to improve care for patients with PI.

## Patient-Centered Systems and Timely Access to Care and Treatment

In countries, which lack a socialized medical system, large or small insurance companies may play a vital role in determining whether patients with PI get equal access to optimal care and treatment. This can be a sticking point for both patients and physician providers, and this is particularly true, for expensive diagnostic testing, and novel treatments, including genetic testing, and gene therapy. Immunoglobulin therapy (considered an “essential” medication for the treatment of primary humoral immunodeficiencies by the World Health Organization, WHO) (6), hematopoietic cell transplantation (HCT), gene therapy, immunomodulatory therapies, enzyme replacement therapy are all necessary treatments for specific disorders, and often prohibitively expensive. It is the belief of the PI medical community that all patients with these diseases should have access to timely and necessary diagnostic testing, specialty care and life-saving and/or curative therapies.

### The US Perspective

The U.S. does not have universal access to healthcare and is one of just a few developed countries left in the world with this glaring deficit. In 2018, 13.7% of adults in the U.S. were not insured. Currently, individual states in the U.S. are providing health insurance for children through the Children's Health Insurance Program (CHIP). The Patient Protection and Affordable Care Act of 2010 sought to improve access to and quality of health care in the US, but many parts of this law have since been repealed. According to the 2017 National Health Interview Survey, 4.5% of people failed to obtain necessary medical care due to cost (*Selected Estimates Based on Data from the National Health Interview Survey*, 2017). Poor financial support for healthcare leads to an inability or delay in patients with PI being diagnosed and treated. Further, the lack of sufficient numbers of subspecialty-trained physicians and the significant demand results in prolonged waiting periods for patients with PI, necessitating many of them to seek emergency care. According to the Commonwealth Fund's 2015 International Profiles of Health Care Systems, over half (52%) of Americans reported they were unable to get a same-day or next-day sick visit appointment with their provider (7). Patients with PI are immunocompromised, thus, they need rapid access to appropriate specialty care, and should not be compelled to seek emergency care with physicians not qualified or well-versed in managing these diseases.

### The Global Perspective

From a global perspective, many of the above issues confront patients with PI, and thus all physicians, specialist societies and organizations, have to work together to ensure that PI patients have appropriate access to specialists (clinical immunologists, and other sub-specialties, including Hematology, Infectious Diseases, Rheumatology, Dermatology, GI, Pulmonology, and Neurology among others), esoteric diagnostic testing, and suitable treatment. Specialist societies in each country or region can direct patients to specialized centers or laboratories that perform immunological testing for primary immunodeficiency, such as the Clinical Immunology Society (CIS) for North America, or the European Society for Immunodeficiencies (ESID), in Europe. They often connect physicians and scientists working in this area, across the globe, providing an educational forum to share information related to clinical care and diagnostic testing.

### Improving Geographic Access to Specialty Care Worldwide

Accessibility to care for patients involves four dimensions: geographic accessibility, availability, financial accessibility, and acceptability (8). The access to care varies across regions of the world with low/middle income countries having a higher population burden, but paradoxically limited access to necessary diagnostic testing and treatment (8). Delays in diagnosis and/or treatment of patients with PI leads to decreased quality of life and preventable morbidity and mortality. Thus, geographic access to PI subspecialty care remains a complex issue worldwide, but more so in many developing and resource-constrained countries. To address these limitations in geographic access, the Asian PID network has established a regional referral network that has helped to facilitate electronic consultation for patients without easy access to a clinical immunologist (9). As telemedicine continues to expand, this model could be adapted more broadly in developing and developed countries to expand care for patients with PI. While, telemedicine could be adapted more broadly in order to aid access concerns, however, there will be limitations to its widespread implementation because of the inaccessibility of patients for a physical exam, and other aspects of healthcare, which require a face-to-face assessment. Since 2014, India, the most populous country in the world, has begun training junior physicians in clinical immunology to facilitate dissemination of knowledge, and train future leaders in PI (10). Care must be available without excessive delays and at convenient times for these patients suffering life-threatening infections, and it also must be affordable and acceptable by local cultural standards (8).

### Expanding Knowledge to Achieve Earlier Diagnosis: The Value of Region-Specific Interventions

Disease awareness in developing countries is an important step to improving in the identification and care of patients with PI (6). In developing countries without proper sanitation or access to antimicrobial agents, it can be difficult to identify infections that are due to an underlying immune dysfunction, making diagnosis of PI a difficult endeavor (6). Therefore, promoting country and region-specific disease registries is essential for expanding the knowledge and known phenotypes of PI in all countries. Current under-represented of PI in registries is exemplified by the fact that although Africa is the second most populous continent, it contains the smallest proportion of estimated cases (11). Awareness of PI in India and through the A-Project in Africa has led to an increase in the volume of PI-related scientific literature being published from these countries (12). Similarly, the Latin American Society for Immunodeficiencies (LASID) has recently established the L-project, which focuses on educating the public/medical professionals on PI, and they have established a fellowship in clinical immunology (13). In Europe, the J Project physician education and clinical research collaboration program, set up in 2004, has established itself as a successful awareness program for primary immunodeficiency in Eastern and Central Europe; and opened new possibilities for the establishment of clinical and genetic databases and joint research (http://www.jprojectnetwork.com/Page/AboutJProject/Ourvision). Another benefit of establishing PI patient registries is the knowledge gained on the unique presentations and diseases that may vary regionally due to genetic diversity and differences in the environment, including endemic microbes. Through such disease-specific registries, information on relatively rarer manifestations of otherwise well-described immunodeficiencies can be obtained, for example, the frequency of arthritis in X-linked agammaglobulinemia (XLA) is ~31% in the Asian population, whereas in Caucasians, it is closer to 10% (9). *Mycobacterium tuberculosis* is endemic in Asia, as a result of which, there is a high rate (41.1%) of Chronic Granulomatous Disease (CGD) patients with tuberculosis infection that is not seen in other areas of the world (9). Finally, in the regions of the world where there is increased consanguinity there is a much higher incidence of rare autosomal recessive diseases that is not apparent in other countries, where this social practice is less common or not practiced (10). Knowledge of these regional differences will enable clinical immunologists to develop region-specific screening, education, diagnosis and treatment, which would benefit the patients.

A significant challenge with diagnostic testing for PI is that most immunological testing is sophisticated, expensive, and often not readily available in developing countries, or certain areas of developed countries (6). In a survey of Latin American laboratories, 72.7% offered testing for post-vaccination pneumococcal titers, while 63.6% offered dihydrorhodamine (DHR) testing for CGD, and 54.5% offered testing for complement. Of these laboratories, only 72.7% had specific quality control and specimen handling requirements (13). In India, most labs do not have the ability to test pneumococcal titers (14). Treatment and diagnosis options between centers in India varies and depends on local expertise and laboratory availability (14). In addition to the challenges in obtaining basic immunology testing in some countries, it is even more difficult to obtain advanced functional, immunophenotyping, and genetic testing. Through their referral network, the Asian PID network has been able to provide genetic testing for patients with suspected PI who would otherwise not have access to genetic testing (9). Lack of access to proper genetic testing in certain areas of the globe is particularly glaring, as the standard of care in many developed countries is moving toward having a molecular diagnosis whenever possible as it has the ability to significantly alter treatment options. In Norway, Iceland, Switzerland, Israel, Qatar, United States, Taiwan, and other countries/regions, newborn screening for severe combined immunodeficiency (SCID) is now universal; and Germany and the Netherlands will soon ensure implementation; however it is not universally performed in most developing countries, though some do have access to this screening. As has been well-established, a delayed diagnosis of SCID can lead to fatal outcomes (15).

As previously mentioned, the WHO considers immunoglobulin (IgG) replacement an essential medication for adults and children with antibody deficiencies (6). However, IgG replacement remains a limited resource worldwide, and is often not available in countries. In Europe for instance, enormous discrepancies exist in care for patients with some countries having no access to immunoglobulin replacement. The European Reference Network (ERN) RITA was established in part to provide access to care for all PID patients. A second wave of ERN-Core centers will focus on Eastern Europe and other unrepresented areas.

While immunoglobulin replacement products are sometimes imported in developing countries, a potential disadvantage of this strategy is that these products from developed countries may not provide antibody protection to pathogens endemic in developing countries (14). Since 2011, there have been great gains in access to immunoglobulin replacement in India due to policies that have led to cost reduction (10). Gene therapy (GT) and HCT for patients with PI is more common and accessible in Europe and North America, but access to these crucial therapies is limited in developing countries (16). While expanded newborn screening for SCID has practical value, the benefit of expanded screening is contingent on having definitive treatments such as HCT and GT readily available, which can also be challenging in the U.S. and Europe, where these remain significantly expensive for many patients.

The creation of continental and regional professional societies focused on PI provides considerable value in supporting physicians and scientists in these areas, through creation of networks that advise and provide practical help for diagnostic testing and treatment. Large geographical inequalities persist and continued progress is needed to achieve ideal patient-oriented care (17). Recently in collaboration with the United States Primary Immunodeficiency Network (USIDNET) Kuwait established a PID registry (18). The ESID registry platform has also been made available to many countries helping to standardize datasets and facilitate research (17). Collaboration between developed and developing nations is key to improving education and access.

Through IAPIDS and the World PI Week campaign, the partnership between PI societies across the globe can be strengthened and used to develop this field with the ultimate goal of serving patients with these rare and complex diseases. In addition, patient advocacy groups (such as the Jeffrey Modell Foundation, the Immune Deficiency Foundation, IPOPI, etc.) play an important role in advocacy, education, and patient support. Developed countries with a more established healthcare infrastructure have a moral imperative to share resources—intellectual and practical—with the ultimate goal of providing the best care for PI patients regardless of where they live on this globe. We have taken the first steps toward this goal with mutual educational opportunities, and we must continue to build on this foundation, as we identify new molecular defects associated with abnormalities of the immune system.

## Author Contributions

NH, KW, and RA wrote the paper and reviewed it.

### Conflict of Interest Statement

The authors declare that the research was conducted in the absence of any commercial or financial relationships that could be construed as a potential conflict of interest.
